# Bilateral severe heterotopic ossification following primary total knee arthroplasty: a case report in Pakistan

**DOI:** 10.1186/s42836-020-00057-1

**Published:** 2021-01-08

**Authors:** Faizan Iqbal, Nouman Memon, Syed Shahid Noor, Nasir Ahmed, Muhammad Farhan Sozera, Arsalan Abro

**Affiliations:** 1 Department of Orthopaedic Surgery, Patel Hospital Street 18, Block 4 Gulshan-e-Iqbal, Karachi, Pakistan; 2 Department of Orthopaedic Surgery, Liaquat National Hospital and Medical College, Karachi, Pakistan

**Keywords:** Complication, Heterotopic ossification, Primary total knee arthroplasty, Treatment

## Abstract

The incidence of unilateral minor heterotopic ossification after primary total knee arthroplasty is still unknown, but bilateral severe heterotopic ossification is rare and has not been reported before. Presented in this report is a 60-year-old female patient who developed bilateral knee pain and stiffness 2 weeks after primary total knee arthroplasty. Her weight was 70 kg and body mass index was 32.2. Preoperatively, she had bilateral varus deformity of both knees. X-rays taken 3 months after surgery revealed bilateral severe heterotopic ossification. The patient had been on non-operative treatment (including anti-inflammatory drugs and physiotherapy). There was a marked improvement 6 months after surgery. This case report showed the non-operative treatment may produce acceptable results for patients with severe bilateral heterotopic ossification after primary total knee arthroplasty, and exerted no influence on the final clinical outcome.

## Introduction

Knee stiffness after primary total knee arthroplasty (TKA) is one of the troublesome problems [1]. New bone formation around the periarticular tissues is called heterotopic ossification (HO), which may be painful and cause a decreased range of motion (ROM) of the knee [1]. The ectopic bone formation around the periarticular tissues is mainly caused by release of osteo-inductive growth factors secondary to the injury to the knee. These growth factors then transform primitive mesenchymal cells into the osteoblastic tissue [2]. The size of HO around the periarticular tissues varies widely, and minor extra bone formation is more common than severe HO [3]. In contrast to total hip arthroplasty, HO after TKA is infrequently encountered. The early studies showed the incidences of HO following TKA were between 4% and 42% [4]. However, the functional impairment is generally minimal. Multiple treatment modalities are available for the treatment of the HO around the knee, including non-operative and operative treatments. Non-operative treatments involve physiotherapy, non-steroidal anti-inflammatory drugs (NSAIDs), manipulation under anesthesia, radiation, *etc*. Operative treatments include arthroscopic release of adhesions, revision TKA, among others [5]. In this case report, we present a patient with severe HO of both knees after primary TKA.

## Case report

**Fig. 1:**
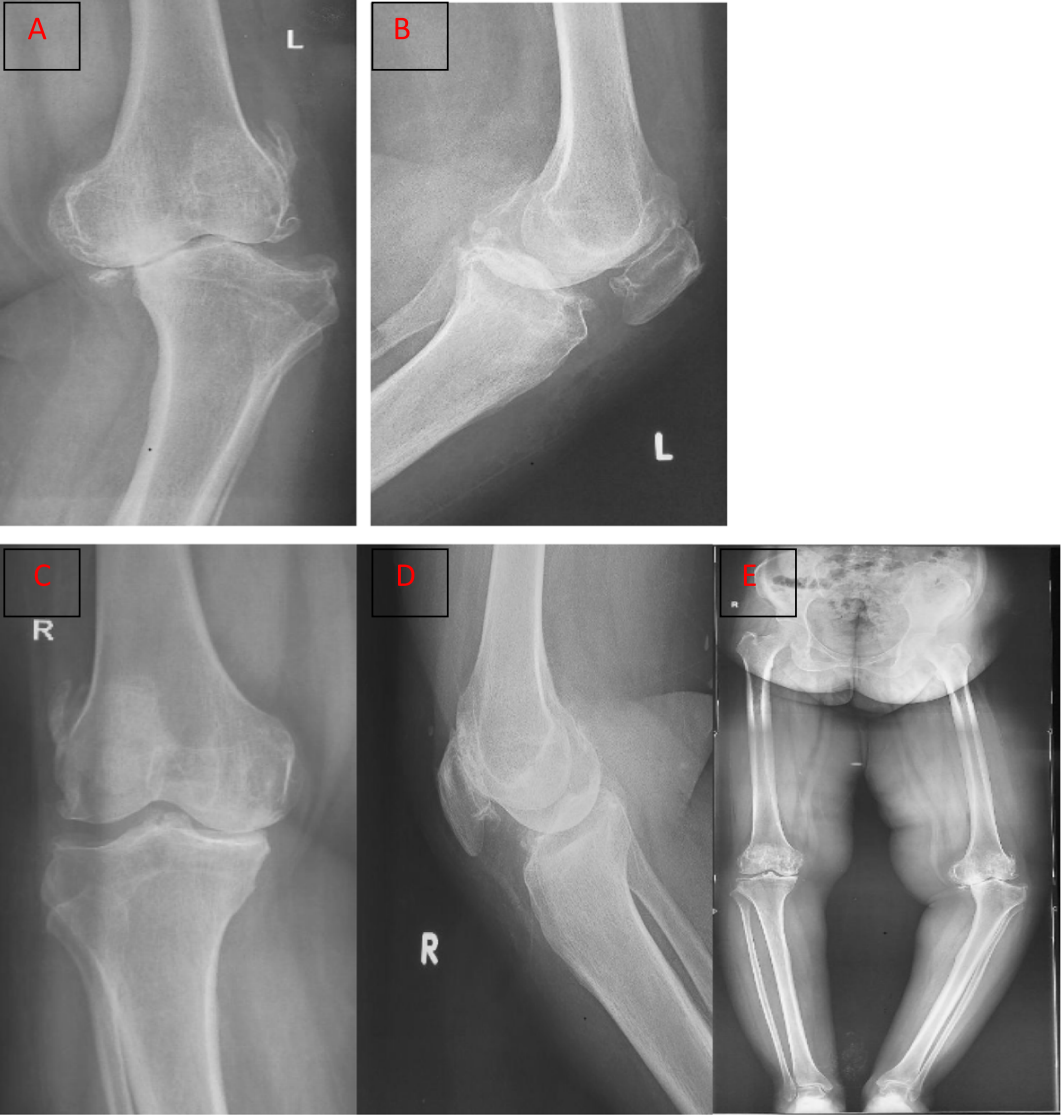
**a**. Anteroposterior (AP) X-ray of left knee showing advanced degenerative changes and severe varus deformity. **b** Advanced degenerative changes on lateral view. **c** Degenerative changes on AP view of right knee. **d** Lateral view of right knee. **e** Full-length standing scanogram showing bilateral varus deformities, which were more severe on the left side

**Fig. 2:**
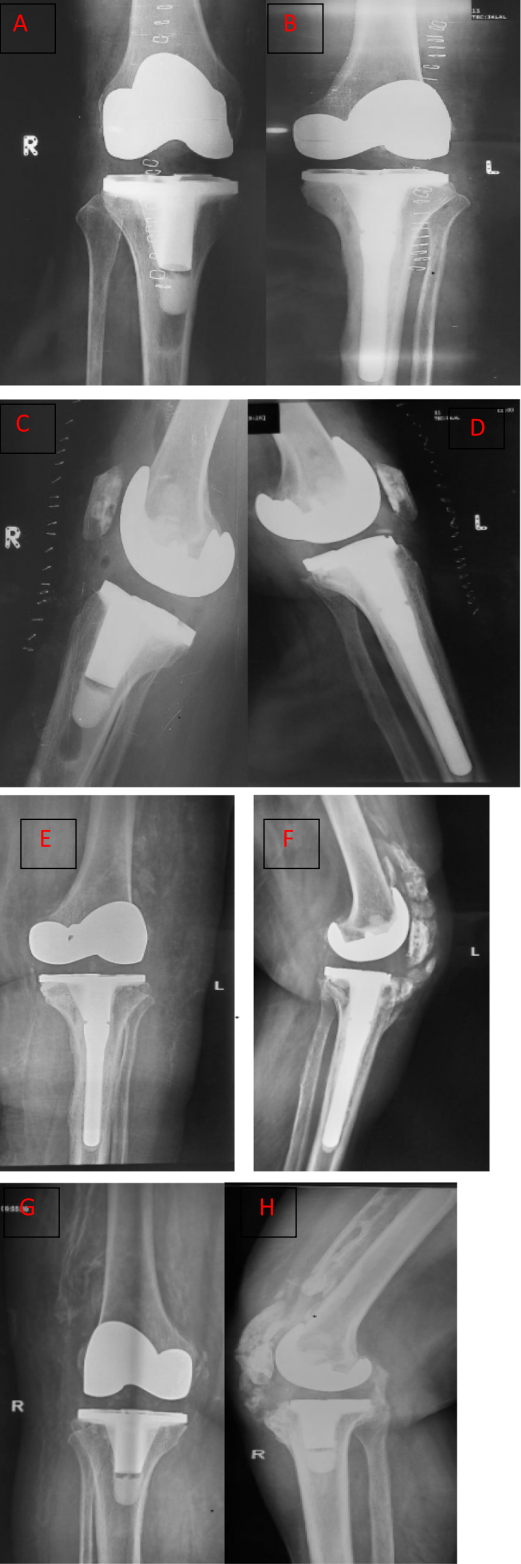
Postoperative AP view of right knee showing the result of cemented total knee arthroplasty (TKA). **b** AP view of left knee showing the result of cemented TKA. **c**. Lateral view of right knee. **d** Lateral view of left knee. **e** AP X-ray showing HO on the lateral aspect of distal femur 3 months after TKA. **f** Lateral view showing HO extending from the tibial tubercle to the proximal tibia. **g** Postoperative AP view of right knee showing HO on the lateral aspect of distal femur. **h** Lateral view of right knee showing severe HO on the anterior aspect of distal femur

**Fig. 3:**
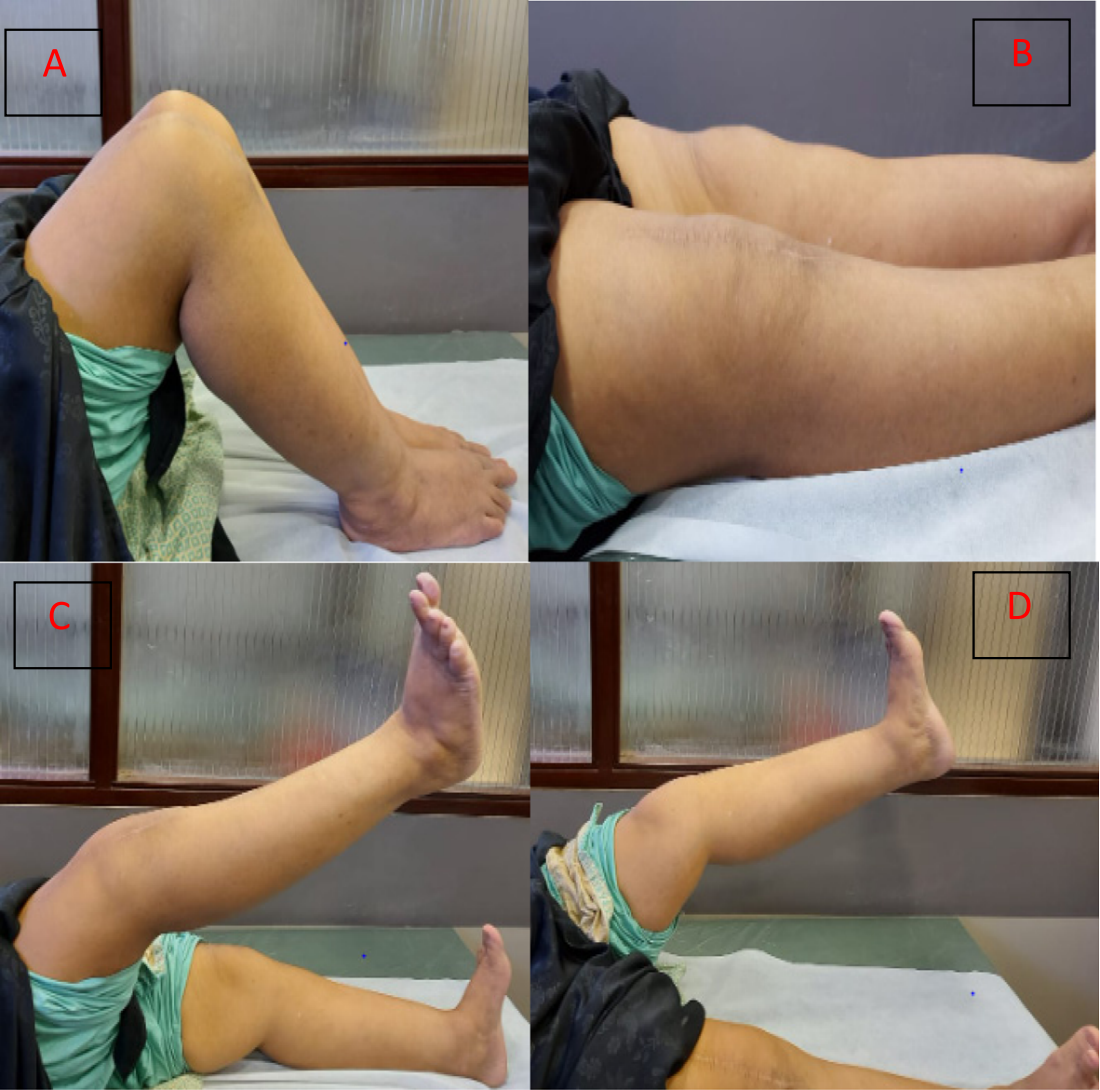
**a**. Maximum flexion of both knees 6 months after surgery. **b**. Full extension. **c**. Active straight leg raise test on the right leg. **d**. The same test on the left leg

A 60-year-old female patient presented to our clinic with complains of knee pain and stiffness following the primary TKA. The patient also had diabetes mellitus, hypothyroidism, rheumatoid arthritis, and depression for over 20 years. She was on insulin, thyroxine, tricyclic anti-depressant drugs, methotrexate, and sulfasalazine. She weighed 70 kg and her body mass index was 32.2. She underwent bilateral cemented TKAs in our hospital because of her advanced knee osteoarthritis for 9 months. Preoperative X-rays showed bilateral knee deformities on the coronal plane. The left knee had severe varus deformity as compared to the right one (Fig. 1a-e). Initial baseline work-up and inflammatory marker tests were performed to rule out underlying infections. The standard medial para-patellar approach was used. The operation was performed by an arthroplasty surgeon with more than 20 years of experience. Initial postoperative period was uneventful, and full-weight-bear mobilization and ROM exercises were allowed since the first postoperative day. Her initial ROM was from 0 to 120 degrees bilaterally. She was discharged safely on the sixth postoperative day. The stitches were removed 2 weeks later. At that time, the ROM of the left and right knees was from + 5 to 110 degrees and from 0 to 120 degrees, respectively. Six weeks after surgery, knee stiffness deteriorated in the right knee as compared to the left knee. The ROM of right and left knees ranged from + 10 to 80 degrees and 0 to 110 degrees, respectively. X-rays revealed HO of both knees, and the left knee was initially more severe. She was advised to receive physiotherapy and NSAIDs. After 3 months, she again presented with severe stiffness of knee joints. The ROM of right knee was from + 10 to 60 degrees, whereas the ROM of left knee was from + 5 to 80 degrees. X-rays taken 3 months after surgery exhibited increased ectopic bone formation on the anterolateral aspect of both knees. She did not suffer from any trauma postoperatively. She was again administered aggressive physiotherapy and NSAIDs. Six months after the surgery, her symptoms gradually improved, and the knee stiffness substantially improved. The ROM of right knee was from 0 to 95 degrees, and left knee from 0 to 100 degrees. X-rays taken immediately after surgery are shown in Fig. 2a-d. X-rays taken 3 months after surgery showed the development of HO (Fig. 2e-h). The ROM of the knees 6 months after surgery are shown in Fig. 3.

## Discussion

HO after total hip arthroplasty is a common complication, but new bone formation after primary TKA is a rare event, and their clinical association is still unknown [4, 5]. HO rarely affects knee function. Therefore, this complication does not attract the attention of knee surgeons [6]. The risk factors for HO formation include male gender, obesity, excessive manipulation and extensive soft tissue dissection during surgery, ankylosing spondylitis, previous injuries to the knee, rheumatoid arthritis, early septic arthritis, preoperative knee deformity, increased lumbar bone marrow density, *etc*. [2, 7] In this case, the patient had severe varus deformity of both knees. Therefore, extensive soft tissue dissection had to be performed intraoperatively to achieve a rectangular gap for soft tissue balancing. The procedure might be the contributing factor to the HO formation, especially in patients with rheumatoid arthritis [8].

Many studies found that HO formation mainly occurred on the anterior aspect of distal femur, which affects the extensor mechanism, thereby affecting knee flexion [5]. Researchers failed to reach a consensus regarding prophylaxis against HO after primary TKA. We didn’t use prophylaxis against HO prior to TKA in this case. An early study showed that radiation and NSAIDs were two modalities used for prophylaxis against HO, especially in high risk cases [2]. Surgery is recommended only when the HO has matured enough around 18 months postoperatively, or when it has interfered with the activities of daily living. The combined therapy of radiation and NSAIDs is indicated in recurrent cases. A comprehensive study must be conducted to reach a consensus regarding the prophylaxis using either radiation or NSAIDs, or both, prior to TKA.

The size of HO also correlates with the overall functional outcome following primary TKA. Dalury *et al*. [9] evaluated 500 patients who underwent cemented TKA. In their series, 8% of patients had type 1 (< 2 cm) new bone formation, 6% had type 2 (2 to 5 cm) new bone formation, and only 1% had type 3 (> 5 cm) new bone formation. The overall incidence of HO was 15%. They found a high incidence in patients who had a preoperative knee deformity of more than 15 degrees, especially in male patients and obese patients. They did not find HO had a major impact on the overall functional outcome following primary TKA irrespective of the size of new bone formation. Non-operative treatments such as physiotherapy and NSAIDs may produce acceptable functional results even in the severe HO.

## Data Availability

Not applicable.

## Abbreviations

TKA: Total knee arthroplasty
HO: Heterotopic ossification
ROM: Range of motion
NSAID: Non-steroidal anti-inflammatory drug
